# The Traditional Chinese Medicine Fuyou Formula Alleviates Precocious Puberty by Inhibiting GPR54/GnRH in the Hypothalamus

**DOI:** 10.3389/fphar.2020.596525

**Published:** 2021-01-21

**Authors:** Guo-liang Bai, Kai-li Hu, Yi Huan, Xing Wang, Lei Lei, Meng Zhang, Chun-yan Guo, Hong-sheng Chang, Li-bo Zhao, Jing Liu, Zhu-fang Shen, Xiao-ling Wang, Xin Ni

**Affiliations:** ^1^ Clinical Research Center, Beijing Children’s Hospital, National Center for Children’s Health, Capital Medical University, Beijing, China; ^2^ State Key Laboratory of Bioactive Substances and Functions of Natural Medicines, Key Laboratory of Polymorphic Drugs of Beijing, Institute of Materia Medica, Chinese Academy of Medical Sciences, Peking Union Medical College, Beijing, China; ^3^ Department of Pharmacology, School of Chinese Materia Medica, Beijing University of Chinese Medicine, Beijing, China

**Keywords:** traditional Chinese medicine, Fy formula, precocious puberty, GPR54/GnRH, hypothalamic

## Abstract

The purpose of this study was to explore the effect of the traditional Chinese medicine Fuyou formula on precocious puberty (PP). The Fy formula may exert an effect in female rats with PP and GT-7 cells through the GPR54/GnRH signaling pathway. To confirm the effect of the Fy formula on PP through the GPR54/GnRH signaling pathway, we first treated GT1-7 cells with the Fy formula and observed changes in the expression of related genes and proteins and in GnRH secretion. Then, we randomly divided young female Sprague-Dawley rats into the control group, model group, leuprorelin group and the Fy formula group. A PP model was established by injection of danazol on postnatal day 5, and the Fy formula was administered on PND15. The time of vaginal opening, the wet weights of the ovary and uterus, serum hormone levels and the expression of hypothalamic-related genes were observed. We found that the Fy formula delayed vaginal opening, decreased the wet weights and coefficients of the ovary and uterus, decreased the levels of serum hormones (E2, follicle-stimulating hormone and luteinizing hormone) and the cellular GnRH level, and downregulated the gene expression of Kiss1, GPR54 and GnRH in the hypothalamus and the gene and protein expression of GPR54 and GnRH in GT1-7 cells. In conclusion, the Fy formula may alleviate PP via the GPR54/GnRH signaling pathway.

## Introduction

Precocious puberty (PP) is one of the most common endocrine diseases in children. PP is commonly defined as puberty that starts before age eight in Chinese girls and age nine in Chinese boys (Bradley et al., 2020), before age 7.5 in African-American and Hispanic girls, and before age eight in Caucasian girls (Herman-Giddens, 2006). The age of pubertal onset based on thelarche has decreased by almost 3 months per decade from 1977 to 2013 (Eckert-Lind et al., 2020). PP is characterized by early onset of puberty, such as early development of secondary sexual characteristics, rapid maturation of bones and acceleration of growth, and early menarche, early closure of the epiphyses, which may lead to poor social adaptability, psychological stress, emotional disorders, risky behaviors, a shorter adult height and future breast and ovarian cancer (Abbasi, 2019; Fu et al., 2020). PP can be divided into central precocious puberty [CPP, gonadotropin-releasing hormone (GnRH)-dependent] and peripheral precocious puberty (PPP, GnRH-independent). The incidence of CPP in China is approximately 1% (Luan et al., 2007). The male to female ratio of patients with PP is approximately 1:5–10 (Teilmann et al., 2005; Dong et al., 2020), and 74% of the cases in girls with CPP are idiopathic (Bradley et al., 2020; Tao et al., 2020). Although the incidence of PP in boys is low, the disorder may be caused by certain serious diseases and should be given attention (Kaplowitz and Bloch, 2016). The onset of puberty is a complex biological process involving numerous factors under the control of neuroendocrine pathways that are regulated by the hypothalamus-pituitary-gonadal (HPG) axis. Tanner stages are used to evaluate pubertal development (Carel and Léger, 2008; Tena-Sempere, 2012). The key step in puberty onset is the early activation of pulsatile GnRH secretion (Ojeda et al., 2006; Latronico et al., 2016). The timing of PP onset depends on genetic and environmental factors, and numerous studies worldwide have shown that the onset of pubertal characteristics varies with race and ethnicity, genetics, environmental conditions, exposure to certain chemicals, geographical location and nutrition (Herman-Giddens et al., 2001; Latronico et al., 2016; Abbasi, 2019). Concerns about PP in girls are extremely common and are frequently raised by parents and other guardians.

Clinical strategies for the treatment of PP in children include a gonadotropin-releasing hormone analog (GnRHa), progesterone and traditional Chinese medicines (TCMs). GnRHa, which has been used clinically for the treatment of CPP treatment for 40 years, mainly contains triptorelin and leuprorelin, which can inhibit early initiation of the hypothalamus-pituitary-gonadal (HPG) axis, delay gonadal and bone development, delay epiphyseal closure and improve adult height (Kletter and Kelch, 1994; Mul and Hughes, 2008; Guaraldi et al., 2016; Fu et al., 2020). However, side effects, such as local hyperlipidemia, erythema, temporary vaginal bleeding, central obesity, body weight and bone mineral density loss, have been reported (Yu et al., 2014). Additionally, some patients require a combination of GnRHa and recombinant growth hormone (rhGH) to achieve the desired effect. However, not all patients who received combination treatment achieved satisfactory height gains (Kohn et al., 1999; Cisternino et al., 2002; Weise et al., 2004; Fu et al., 2020). CPP is diagnosed based on the GnRHa stimulation test (Carel et al., 2009). The assessment and management of this disease remain a challenge for pediatric endocrinologists (Latronico et al., 2016), and the Pediatric Endocrinology Society has suggested that the combination of GnRHa and rhGH should not be recommended as routine treatment because of its high cost and the lack of large-scale randomized clinical trials evaluating the safety and effectiveness of this combination therapy (Carel et al., 2009).

TCM formulas have played an important role in the modernization of TCM. However, there are many TCM compound preparations, which ingredients of which are complex, and most of them contain many minerals and animal medicinal materials. Some drugs have various bad tastes and smells, such as a bitter taste, astringent taste or sour taste. Therefore, the chemical composition of TCM formulas is more complex than that of a single herbal medicine.

TCM practitioners in China have used oral herbs to treat many pediatric diseases for thousands of years. CPP is rarely reported in ancient literature, probably because it is not very common. However, some Chinese herbal formulas, such as the Nourishing (Yin) Removing (Fire) Chinese herbal mixture (Sun et al., 2010; Wang et al., 2014b; He et al., 2018), Zhi Bai Di Huang Wan (Anemarrhena, Phellodendron, and Rehmannia Pill) (Cheng et al., 2013), Jia Wei Xiao Yao San (Supplemented Free Wanderer Powder) (Yamada and Kanba, 2002), Long Dan Xie Gan Tang (Gentian Liver Draining Decoction) (He et al., 2015), Xiang Sha Liu Jun Zi Tang (Costustoot and Amonum Six Gentlemen Decoction) (Xiao et al., 2012), Cang Er Zi San (Xanthium Powder) (Yang and Yu, 2008), Wen Dan Tang (Gallbladder-Warming Decoction) (Wang et al., 2014a), and Ma Zi Ren Wan (Hemp Seed Pill) (Hu et al., 2015), have been reported to alleviate PP.

Fuyou (Fy) formula is an in-hospital preparation that clears the liver, nourishes yin and removes fire, as described by pediatric gynecologists at Beijing Children’s Hospital on the basis of TCM pathogene-sis and the physical characteristics of PP. The salt Anemarrhena and Radix Rehmanniae are the primary components of the Fy formula, which contains 11 TCMs that act together to clear the liver and disperse kont, nourish yin and remove fire. Our team found that clinical application over the past 20 years has suggested that the formula can control the early symptoms of PP in girls, reduce the size of the mammary glands, decrease the level of estrogen and delay the rate of bone maturation without obvious adverse reactions (Huili Liu and Liu, 2009; Pan Yu-chen and Liu, 2019).

In the present paper, we treated GT1-7 cells and young Sprague-Dawley (SD) rats with danazol-induced PP with the Fy formula and analyzed clinical data from a 1-year period. We confirmed the efficacy of the Fy formula for the treatment of PP and preliminarily identified the underlying mechanism. As children are a vulnerable group, it is not feasible to conduct randomized placebo-controlled clinical trials on these patients. Therefore, we compared clinical data before and after treatment.

## Materials and Methods

### Chemicals and Reagents

Dulbecco’s modified Eagle’s medium (DMEM, 10–013-CV) and fetal bovine serum (FBS, 35–081-CVR) were obtained from Corning (NY, United States). Trypsin (0.25%) was purchased from Gibco (Grand Island, NY, United States). Isopropanol and chloroform were purchased from Sinopharm Chemical Reagent Co., Ltd. (Shanghai, China). Penicillin/streptomycin were obtained from Sigma-Aldrich (St. Louis, MO, United States). The other reagents were of analytical grade and had a purity of 99.5% or higher. Kisspeptin-10 (kp-10, 45–54) was purchased from Anaspec (San Jose, United States). Danazol was obtained from A&D Technology Corporation (Beijing, China). Leuprorelin acetate microspheres for injection were purchased from Livzon (Zhuhai, China). Leuprolide acetate was purchased from TargetMol (MA, United States). Rabbit anti-GPR54, rabbit anti-kiss1, and rabbit anti-HSP90A antibodies were all purchased from Absin Bioscience Inc. An anti-GnRH antibody was purchased from Lifespan (LS-C294315). Rabbit anti-Erk1/2 (4,695) and anti-pErk1/2 antibodies (4,370) were purchased from CST. ELISA kits for follicle-stimulating hormone (FSH), luteinizing hormone (LH), and estradiol (E2) were obtained from Cloud-Clone Corp. A mouse GnRH ELISA kit was obtained from mIbio (mI701852). The TCM standards Mangiferin (serial number: 111607, purity: 98.1%), Paeoniflorin (serial number: 110736, purity: 97.4%) and Gentiopicrin (serial number: 110770, purity: 98.2%) were purchased from the National Institutes for Food and Drug Control.

### Preparation of the Traditional Chinese Medicine Fuyou Formula

The Fy formula was prepared by combining the following 11 medicinal plants: 10 g each of Sheng Di Huang [*Rehmannia glutinosa* (Gaertn.) DC, *Orobanchaceae*], Yan Zhi Mu [*Anemarrhena asphodeloides* Bunge, *Asparagaceae*], Cu Bie Jia (*Trionycis carapax*, *Trionyx sinensis* wiegmann), Xia Ku Cao (*Prunellae spica*, *Prunella vulgaris* L.), Mu Dan Pi (*Paeonia × suffruticosa* Andrews, *Paeoniaceae*), and Chao Mai Ya [*Hordeum vulgare* L. (*Poaceae*), *Poaceae*]; 6 g each of Xuan Shen (*Scrophularia ningpoensis* Hemsl, *Scrophulariaceae*), Di Gu Pi (*Lycium chinense* Mill, *Solanaceae*), Long Dan Cao (*Gentiana scabra* Bunge, *Gentianaceae*), and Ze Xie [*Alisma plantago-aquatica* subsp. Orientale (Sam.) Sam, *Alismataceae*]; and 3 g of Huang Bai (*Phellodendron chinense* C.K.Schneid, *Rutaceae*). The TCM compounds are listed in Table 1. The Fy formula dry paste powder was provided by Professor Ni Jian from Beijing University of Chinese Medicine after process optimization. The optimized process was as follows: 10 times the amount of water in the whole formulation was added, vinegar turtle was added for 30 min, the remaining 10 TCMs were added, the solution was extracted 3 times for 1 h each, the three decoctions were combined, and the extract was concentrated (at 60 °C) to obtain a dry paste powder. Powdered Fy (40 mg) was dissolved in 1 ml of distilled water and subjected to for ultrasonic treatment for 30 min (power: 250 W, frequency: 40 KHz). The solution was filtered with a 0.22 µm microporous filter membrane, and a sample was used to treated cells. The main components of the Fy formula were determined by high performance liquid chromatography (HPLC).

**TABLE 1:** Composition of the Fy formula.

Chinese name	Scientific name	Family	Lot No	Place of origin	Parts of plant used (voucher numbers)
Sheng Di Huang	*Rehmannia glutinosa* (Gaertn.) DC	Plantaginaceae	20181101	Henan, China	Dried root tuber (C18–11001)
Yan Zhi Mu	*Anemarrhena asphodeloides* Bunge	Asparagaceae	20180915	Hebei, China	Dried rhizome (C18–09358)
Cu Bie Jia	*Carapax Trionycis*	Trionyxsinensis Wiegmann	20180903	Hubei, China	Carapace (C18–09047)
Xia Ku Cao	*Prunella vulgaris L*	Lamiaceae	20181006	Jiangsu, China	Dried aerial parts (C18–10063)
Mu Dan Pi	*Paeonia x suffruticosa Andrews*	Paeoniaceae	20181008	Anhui, China	Dried root bark (C18–10127)
Chao Mai Ya	*Hordeum vulgare* L	Poaceae	20180912	Hebei, China	Fruit (C18–09270)
Xuan Shen	*Scrophularia ningpoensis* Hemsl	Scrophulariaceae	20180907	Zhejiang, China	Dried root tuber (C18–09126)
Di Gu Pi	*Lycium chinense* Mill	Solanaceae	20181112	Henan, China	Dried root bark (C18–11315)
Long Dan Cao	*Gentiana* scabra Bunge	Gentianaceae	20180514	Yunnan, China	Dried roots and rhizomes (C18–05490)
Ze Xie	*Alisma plantago-aquatica* L	Alismataceae	20181005	Fujian, China	Dried tuber (C18–10036)
Huang Bai	*Phellodendron chinense* C.K.Schneid	Rutaceae	20181027	Heilongjiang, China	Dried bark (C18–10661)

### Animals

At postnatal day (PND)3, female SD rats and their mothers were obtained from SPF Biotechnology Co., Ltd. [Beijing, license no: SYXK (Beijing) 2016–0038]. The SD rats were housed at a constant temperature (23 ± 2°C) on a 12-h light:12-h dark cycle in an SPF animal room. The animals were provided food and water ad libitum and acclimated for three days before the experiment. All animal experiments were performed in strict compliance with Chinese guidelines, including the standards for Laboratory Animals (GB14925–2001) and the Guideline on the Humane Treatment of Laboratory Animals (MOST 2006), and all animal procedures were approved by the Beijing Administration Office for Laboratory Animals (approval number: SCXK-Beijing-2014-0004).

### Animal Grouping and Drug Administration

The animals were randomly divided into four groups: the control group, model group, positive control (leuprorelin) group, and Fy formula (Fy) group. At PND5, the rats in the three groups were given a single subcutaneous injection of 300 µg/25 µL danazol (ethylene glycol: ethanol = 1:1, v/v) except the rats in the control group, which were given a subcutaneous injection of 25 µL of glycol/ethanol (Morishita et al., 1993; Ju et al., 2019). The rats in the leuprorelin group were subcutaneously injected with 100 μg/kg leuprorelin. The rats in the Fy group were given dry ointment powder solution by intragastric administration every day, and the rats in the control and model groups were given the same amount of normal saline. The vaginal opening was observed at PND20, and the vaginal exfoliated cells of the rats that exhibited vaginal opening were examined by smear. The rats that exhibited vaginal opening were sacrificed at diestrus after a complete estrous cycle, while the remaining rats were sacrificed at the same time point. All rats were anesthetized by intraperitoneal injection of 2% pentobarbital sodium. Blood samples were collected from the abdominal aorta before execution, and the serum was separated after centrifugation (3,500 rpm, 20 min, 4°C) and preserved at −80°C for analysis of the serum hormone level. After the rats were sacrificed, the hypothalamus was removed, frozen in liquid nitrogen and stored at −80°C for further analysis. The uterus and ovaries were weighed to assess the organ coefficients (expressed as mg/100 g body weight) and fixed in formalin for hematoxylin and eosin (H&E) staining.

The Fy formula dose was calculated according to the clinical dosage administered to 6-year-old girls according to the following formula:

$$d_{B}=d_{A}\times R_{B}/R_{A}\times {\left(W_{A}/W_{B}\right)}^{1/3}$$

with d_B_ representing the animal/human body weight dose, d_A_ representing the known human/animal body weight dose, W_A_ and W_B_ representing known human and animal weights, respectively, and R_A_ and R_B_ representing known human/animal body shape coefficients, respectively. Every two days, the animals were weight, and the dose was recalculated.

### Hormone Assay

The serum concentrations of FSH, LH and E2 were measured by kits for ELISA, which is a competitive inhibition enzyme immunoassay technique, purchased from Cloud-Clone Corp. (Wuhan, China). The samples were measured in duplicate, and all the samples were measured together. The minimum detectable concentration of FSH was less than 1.11 ng/ml, and the intra-assay and inter-assay coefficients were less than 10 and 12%, respectively. The minimum detectable concentration of LH was less than 37.59 pg/ml, and the intra-assay and inter-assay coefficients were less than 10 and 12%, respectively. The minimum detectable concentration of E2 was less than 4.45 pg/ml, and the intra-assay and inter-assay coefficients were less than 10 and 12%, respectively.

### GT1-7 Cell Culture and Maintenance

Mouse GnRH-producing hypothalamic GT1-7 neurons (provided by Pamela L. Mellon, University of California San Diego) were cultured in complete fresh DMEM (Corning) containing 10% (v/v) heated-inactivated FBS and 1% antibiotics (100 U/ml penicillin/streptomycin) (Liu et al., 2018) under humidified conditions at 37°C in a 5% CO_2_-containing atmosphere. The medium was changed every 3 days, and the cells were passaged at 90% confluency for subsequent experiments. The cell line was confirmed to be free of *mycoplasma* contamination by PCR and culture. When the cells reached approximately 85% confluence, they were washed twice with PBS, digested with 0.25% trypsin-EDTA and resuspended in a single-cell suspension. Then, 1.0 × 10^6^ cells were seeded in a 6-well plate in 2 ml of medium containing 10% FBS for 24 h, and 2.0 × 10^4^ cells were seeded in a 96-well Corning plate in 100 µL of medium containing 10% FBS for 24 h. Then, the cells were administered different treatments in serum-free medium (SFM) for 24 h depending on the experiment.

### Cell Counting Kit-8 Assay

Cell counting kit-8 (CCK-8) was purchased from MCE. To assess the toxicity of the Fy formula, GT1-7 cells were seeded in 96-well tissue culture plates at a density of 2.0 × 10^4^ cells per well in 100 µL of medium. After overnight incubation, the cells were incubated with 1.25, 2.5, 5, 10, 20, or 40 μg/ml Fy formula in SFM for 24 h at 37°C, and then 10 µL of CCK-8 solution (MCE, HY-K0301) was added to the cells. Following an incubation period of 1–4 h, the optical density (OD) was measured at 450 nm using an ELISA plate reader.

### RNA Extraction and Quantification

Total RNA was extracted from frozen GT1-7 cells and hypothalamic tissue using TRIzol reagent (Invitrogen, United States). Purified total RNA was solubilized in diethylpyrocarbonate (DEPC)-treated water (Beyotime, China), and the concentration was determined using a Biodropsis BD-2000 spectrophotometer (OSTD Beijing Co., Ltd., China) at 260/280 or 260/230 nm. Two micrograms of total RNA was used as a template for reverse transcription using a cDNA synthesis kit (TransGen Biotech, AT311, TransScript One-Step gDNA Removal and cDNA Synthesis SuperMix), and the synthesized first-strand cDNA was stored at *−*80°C until use. The expression of each gene was quantified by real-time PCR (TransGen Biotech, AQ141, TransScript Tip Green qPCR SuperMix) using the following protocol: 1 cycle at 94°C for 30 s followed by 45 cycles at 94°C for 5 s, 60°C for 15 s and 72°C for 10 s. Briefly, the ΔCt value of each sample normalized to the internal control was calculated using the following equation: ΔCt = Ct (gene) − Ct (β-actin). To assess differences in expression between experimental and control conditions ΔΔCt was calculated with the following formula: ΔΔCt = ΔCt (sample) − ΔCt (control). The results are expressed as fold change in expression calculated by the 2^ΔΔCt^ method, and the levels of the of transcripts were normalized to the level of β-actin.

Single-stranded oligodeoxynucleotide primers for amplification of each gene were designed based on PubMed searches and were synthesized by Invitrogen (Beijing, China). The sequences of these primers are listed in Table 2.

**TABLE 2:** Primer sequences for quantitative real-time polymerase chain reaction.

Gene	Forward primer (5′-3′)	Reverse primer (5′-3′)
β-actin (mouse and rat)	ACT​CTT​CCA​GCC​TTC​CTT​C	ATC​TCC​TTC​TGC​ATC​CTG​TC
GnRH (mouse)	GGG​AAG​ACA​TCA​GTG​TCC​CAG	CTC​GAG​CTT​CCG​TTG​GTA​GG
GPR54 (mouse)	CTG​TCA​GCC​TCA​GCA​TCT​GG	AGC​AGC​GGC​AGC​AGA​TAT​AG
Erα (mouse)	AAG​ACG​CTC​TTG​AAC​CAG​CA	CGA​GTT​ACA​GAC​TGG​CTC​CC
Erβ (mouse)	AAG​ACG​CTC​TTG​AAC​CAG​CA	CGA​GTT​ACA​GAC​TGG​CTC​CC
GnRH (rat)	TGG​TAT​CCC​TTT​GGC​TTT​CAC​A	TGA​TCC​TCC​TCC​TTG​CCC​AT
Kiss1 (rat)	CAA​TGG​TCT​GAA​CTG​CCC​AC	CAC​AGG​TGC​CAT​TTT​TGC​CA
GPR54 (rat)	CAA​CCT​GCT​GGC​CCT​ATA​CC	CTAGCAGCTGCAGGGCG

GnRH, gonadotropin-releasing hormone; Kiss1, Kiss-1 metastasis suppressor; GPR54, G-protein coupled receptor 54; Erα, estrogen receptor alpha; Erβ, estrogen receptor beta.

### GnRH mRNA Expression

GT1-7 cells were plated in 6-well plates at a density of 1.0 × 10^6^ cells per well in 2 ml of complete medium and incubated overnight. Then, the medium was replaced with SFM, and the cells were treated with kp-10 at final concentrations of 10^−11^, 10^−9^, 10^−8^, 10^−7^, 10^−6^ or 10^−5^ M for four or 24 h. The optimal concentration and duration for GnRH activation were determined. Cells were co-incubated with the Fy formula and the optimal concentration of kp-10, and the effect of the Fy formula on the GnRH gene was investigated.

### GnRH Secretion

GT1-7 cells were plated into 96-well plates, and after 24 h, the cells were treated with kp-10 at final concentrations of 10^−11^ to 10^−5^ M for four or 24 h. To study the effects of the Fy formula in the presence of kp-10, cells were treated with 5 or 10 μg/ml Fy formula and 10^–9^ M kp-10 for 24 h. Then, the 96-well plate was centrifuged (1,000 rpm, 10 min), and the supernatant was transferred to a new 96-well plate for the determination of GnRH levels. RIPA lysis was added to the cells to adjust the protein concentration.

### Protein Extraction and Western Blot Analysis

Western blot analysis was performed as previously described (Wang et al., 2017). To assess protein levels, GT1-7 cells were plated into 6-well culture plates at a density of 1.0 × 10^6^ cells per well in 2 ml of SFM and incubated overnight. Then, the GT1-7 cells were incubated with noncytotoxic Fy formula concentrations for 24 h. Then, they were lysed in RIPA lysis buffer supplemented with protease and phosphatase inhibitor cocktail (Applygen Technologies, Inc., China), and the BCA assay was used for quantitative analysis. Equal amounts of protein samples were separated by electrophoresis on 10% sodium dodecyl sulfate polyacrylamide gels and transferred to polyvinylidene fluoride membranes. The membranes were blocked with 5% BSA in Tris-buffered saline containing 0.05% Tween 20 (TBS) for 1 h at room temperature and then incubated with primary antibody at 4°C in blocking buffer. The membranes were washed three times for 10 min with TBST and then incubated for 1 h at room temperature with a horseradish peroxidase-labeled secondary antibody (1:2,000, ZSGB-BIO, Inc., China). An enhanced chemiluminescence detection system was used to visualize the signal. Protein band densities were analyzed by Gel-Pro Analyzer 3.1 software, and the protein expression of β-actin was used as an internal standard.

### Histopathological Evaluation of the Uterus

Uteri harvested from female rats were fixed in 4% paraformaldehyde, embedded in paraffin, and cut into 4-mm slices using a slicing machine. The degree of pathological improvement in the uterus was evaluated by H&E staining.

### Statistical Analysis

All results are presented as the means ± SDs. Differences were analyzed by one-way ANOVA, and the data were plotted with GraphPad 7 (GraphPad Software, San Diego, CA). Differences were considered statistically significant when *p* < 0.05.

## Results

### Multicomponent Quantification of the Fuyou Formula High Performance Liquid Chromatography

**FIGURE 1:**
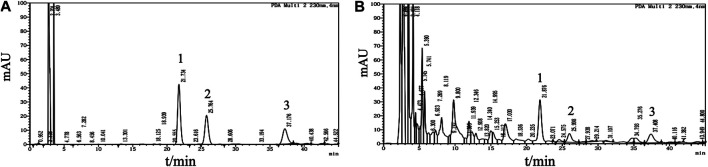
Multicomponent quantification of the Fy formula. **(A)** Chromatogram of the mangiferin, paeoniflorin and gentiopicrin standard mixture. Peak one corresponds to gentiopicrin, peak two corresponds to mangiferin, and peak three corresponds to paeoniflorin. Mangiferin (1.5 mg), paeoniflorin (3 mg) and gentiopicrin (9 mg) were accurately weighed and placed in a 20 ml volumetric flask. The mixture was diluted with methanol to the mark on the flask and shaken to obtain a mixed standard mother liquor. Then, 1.5 ml of the mixed standard mother solution was added to a 5 ml volumetric flask and diluted with methanol to the mark on the flask to obtain the reference solution. **(B)** HPLC profile of the Fy formula. The powered compound extract (0.5 g) was accurately weighed and placed in a stoppered conical flask. Then, 25 ml of methanol was added, and the flask was tightly stoppered and weighed. The compound was subjected to ultrasonic treatment for 30 min, methanol was added until the original weight was obtained, and the compound was shaken well and filtered.

Analyses were performed using the LC Solution Chromatography workstation (Shimadzu Corporation, Kyoto, Japan). Chromatography was carried out at 30°C on an Angilent Zorbax C18 column (4.6 mm × 150 mm, 5 μm; Agilent Technologies, Santa Clara, CA, United States). The mobile phase consisted of acetonitrile and 0.2% (v/v) acetic acid-water solution (v/v = 9:91) for isocratic elution at a flow rate of 1.0 ml/min. The signal was monitored at wavelengths of 258 and 230 nm. The chromatograms of the standard mixture and Fy formula are presented in Figure 1.

### The Fuyou Formula Delays Vaginal Opening in Female Rats With Precocious Puberty

**FIGURE 2:**
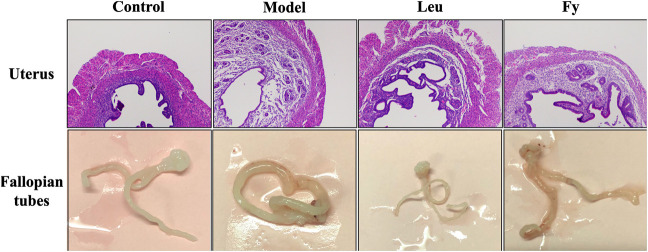
Effects of the Fy formula on uterine wall thickness and fallopian tubes. The uterine wall was thicker in the model group than the control group and returned to normal thickness after administration of the Fy formula. The fallopian tubes of the model were thicker than those of the control group, and the fallopian tubes returned to normal thickness after administration of the Fy formula. However, in the leuprorelin group, the fallopian tubes were significantly thinner than those in the model group.

In the model group, vaginal opening was observed at PND21 and was completed vaginal opening by PND26. Vaginal opening occurred at PND25 and PND23 in the leuprorelin and Fy groups, respectively. Complete vaginal opening occurred in six rats in the leuprorelin group, six rats in the Fy group, one rat in the control group and all rats in the model group. The mean time of vaginal opening in the Fy group (25.7 ± 2.1) was obviously delayed compared to that in the model group (22.9 ± 1.7, *p* < 0.05, Figure 2A).

### The Fuyou Formula Inhibits Sex Organ Development in Rats with Danazol-Induced Precocious Puberty

**FIGURE 3:**
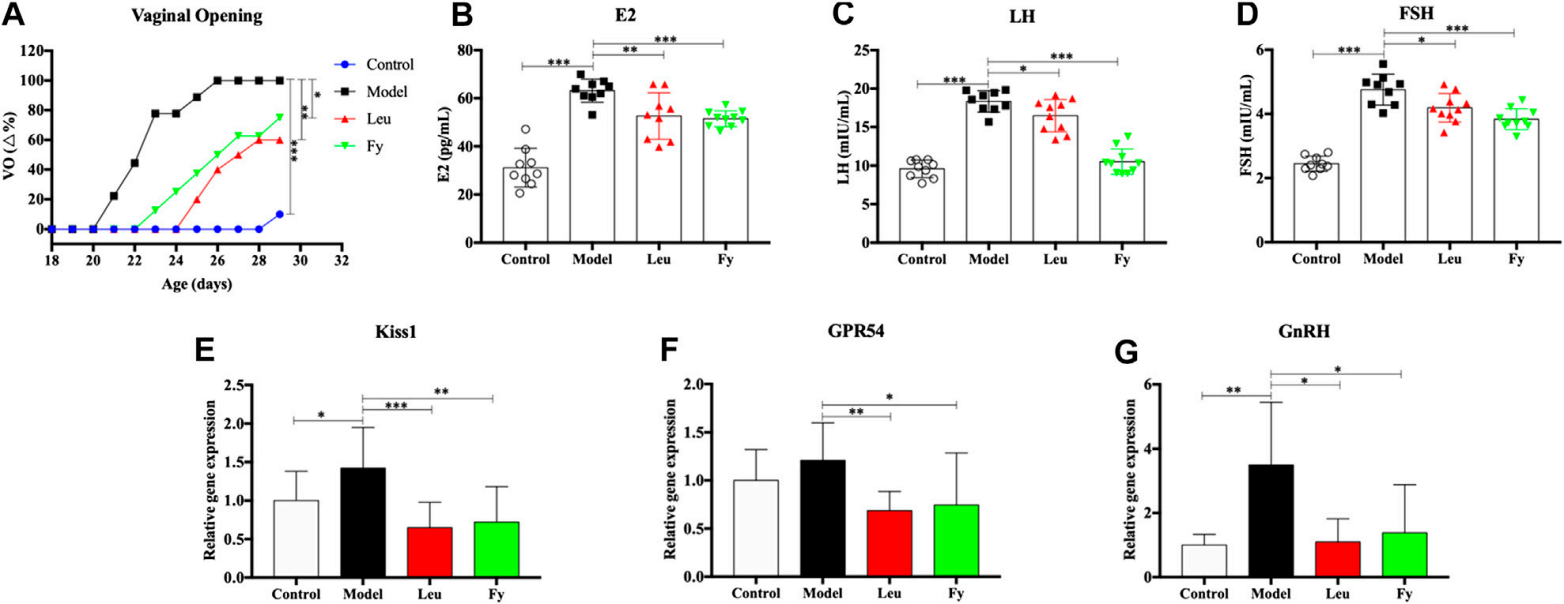
Effects of the Fy formula on vaginal opening **(A)**, serum levels of the hormones E2, LH and FSH **(B–D)** and gene expression in the hypothalamus **(E–G)** in female rats with PP. **(A)** The day of vaginal opening was earlier in the model group than in the control group and was significantly delayed by leuprorelin and the Fy formula. **(B–D)** The serum levels of the hormones E2, LH and FSH were significantly higher in the model group than the control group and lower in the leuprorelin and Fy formula groups than the model group. **(E–G)** The gene expression of Kiss1 and GnRH, but not GPR54, in the hypothalamus was higher in the model group than the control group, and the expression of all three genes was decreased after administration of leuprorelin and the Fy formula. *p < 0.05, **p < 0.01, ***p < 0.001 vs the model group.

As shown in Table 3, the wet weights of the uterus and ovaries, organ coefficients and uterine wall thickness in the model group were obviously lower than those in the control group, while the wet weights of the organs, organ coefficients and uterine wall thickness were significantly lower in the leuprorelin and Fy groups than the model group (Figure 3). We found that the fallopian tubes of the model group were obviously thicker than those of the control group and that the thickness of the fallopian tubes became more similar to that of the control group after administration of the Fy formula. The fallopian tubes of the leuprorelin group were thinner than those of the model group (Figure 3), indicating that leuprorelin affects the normal development of rats and should be combined with growth hormone, as described in the literature (Weise et al., 2004).

**TABLE 3:** Uterine and ovarian wet weights, organ coefficients and uterine wall thickness (n = 8–10).

Group	Wet weight of the uterus (g)	Organ coefficient of the uterus (10^−4^)	Wet weight of the ovary (g)	Organ coefficient of the ovary (10^−4^)	Uterine wall thickness (mm)
Control	0.0962 ± 0.0230	10.81 ± 2.62	0.0406 ± 0.0053	4.58 ± 0.66	4.62 ± 2.10
Model	0.1841 ± 0.0496^###^	19.88 ± 5.21^###^	0.0508 ± 0.0069^##^	5.53 ± 1.08^#^	7.35 ± 1.98^#^
Leu	0.0402 ± 0.0083^***^	4.33 ± 0.67^***^	0.0149 ± 0.0023^***^	1.62 ± 0.26^***^	3.28 ± 1.90^***^
Fy	0.1363 ± 0.0253^*^	15.00 ± 3.09^*^	0.0391 ± 0.0059^**^	4.30 ± 0.66^*^	4.84 ± 1.27^*^

### The Fuyou Formula Decreases Blood Hormone Levels and Inhibits GPR54 Expression in Rats with Danazol-Induced Precocious Puberty

ELISA was employed to evaluate the serum levels of the hormones E2, LH and FSH in PP rats. The results suggested that the levels of hormones E2, LH and FSH were higher in the model group than in the control group (*p* < 0.001). However, the levels of E2, LH and FSH in the leuprorelin and Fy formula groups were obviously lower than those in the model group (Figures 2B–D).

We observed that the gene expression of kiss1, GPR54 and GnRH was obviously lower in the hypothalamic of SD rats with danazol-induced PP than in the hypothalamic of control rats and that the Fy formula and leuprorelin significantly reduced the expression of kiss1, GPR54 and GnRH genes compared with that in the model group (Figures 2E–G). However, there was no significant difference in GPR54 gene expression between the control and model groups.

### The Fuyou Formula Downregulates the Expression and Secretion of GnRH in Hypothalamic GT1-7 Neurons

The effect of the Fy formula on the viability of GT1-7 cells was compared with that of vehicle treatment. The maximum noncytotoxic concentration of the Fy formula was 10 μg/ml (Supplementary Figure S1). To ensure that the Fy formula was administered at a sufficient concentration to exerts the desired effects, the final treatment concentrations for all subsequent experiments in GT1-7 cells were 5 and 10 μg/ml. Changes in GnRH mRNA expression in GT1-7 cells after treatment with different concentrations of kp-10 for 4 or 24 h were evaluated by RT-PCR. The expression of GnRH mRNA was the highest when GT1-7 cells were treated with kp-10 at a concentration of 10^*−*9^ M for 24 h (Supplementary Figure S2).

**FIGURE 4:**
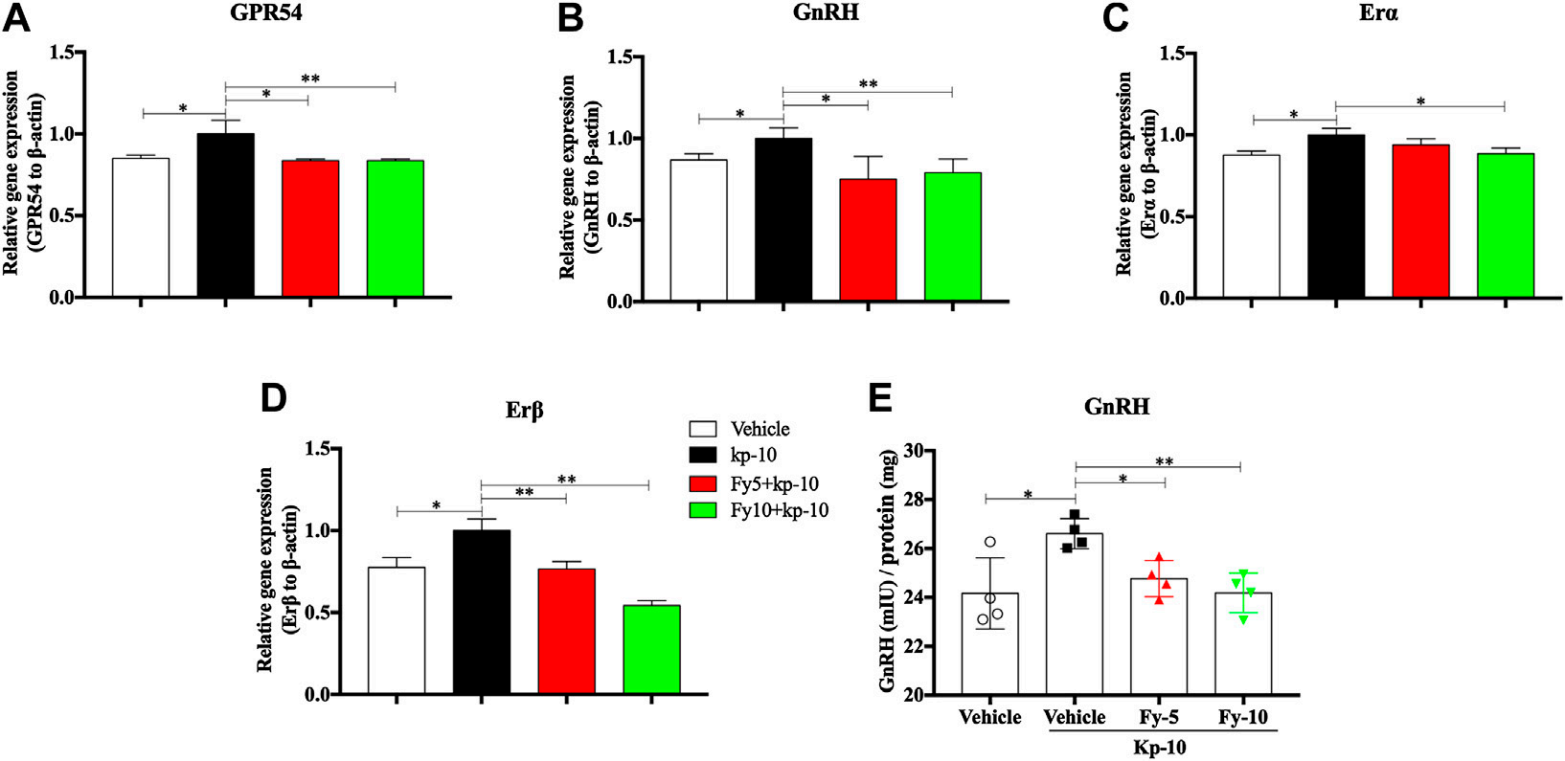
Effects of the Fy formula on GnRH mRNA expression **(A–D)** and secretion in GT1-7 cells **(E)**. GT1-7 cells were divided into the vehicle group (untreated GT1-7 cells), kp-10 group (GT1-7 cells treated with kp-10), and Fy + kp-10 group (GT1-7 cells treated with 5 or 10 μg/ml Fy formula and kp-10 for 24 h). **(A–D)** The gene expression of GPR54, GnRH, Erα and Erβ was higher in the kp-10 group than the vehicle group and lower in the Fy formula group than the vehicle group. **(E)** Cells treated with kp-10 (24 h) exhibited increased secretion of GnRH and decreased secretion when coincubated with the Fy formula. *p < 0.05, **p < 0.01, ***p < 0.001 vs. the kp-10 group.

To further evaluate the mechanism by which the Fy formula affects the release of GnRH in GT1-7 cells via the GPR54/GnRH signaling pathway, the gene expression of GPR54, GnRH, estrogen receptor α (Erα) and estrogen receptor β (Erβ) in GT1-7 cells treated with kp-10 was detected by RT-PCR. The RT-PCR results showed that compared with vehicle treatment, treatment with 10^−9^ M kp-10 for 24 h significantly upregulated the gene expression of GPR54, GnRH, Erα and Erβ (Figures 4A–D) in GT1-7 cells. Additionally, the expression of GnRH, GPR54, and Erβ in the Fy + kp-10 groups, except for Erα expression in the Fy5 + kp-10 group, was significantly lower that than in the kp-10 group.

To evaluate the effect of the Fy formula on GnRH secretion, GT1-7 cells were treated with five or 10 μg/ml Fy formula. Kp-10 significantly elevated GnRH secretion; however, compared with kp-10, the Fy formula significantly decreased GnRH secretion in a concentration-dependent manner (Figure 4E).

### The Fuyou Formula Inhibits the GPR54/GnRH Pathway in GT1-7 Hypothalamic Neuronal Cells

**FIGURE 5:**
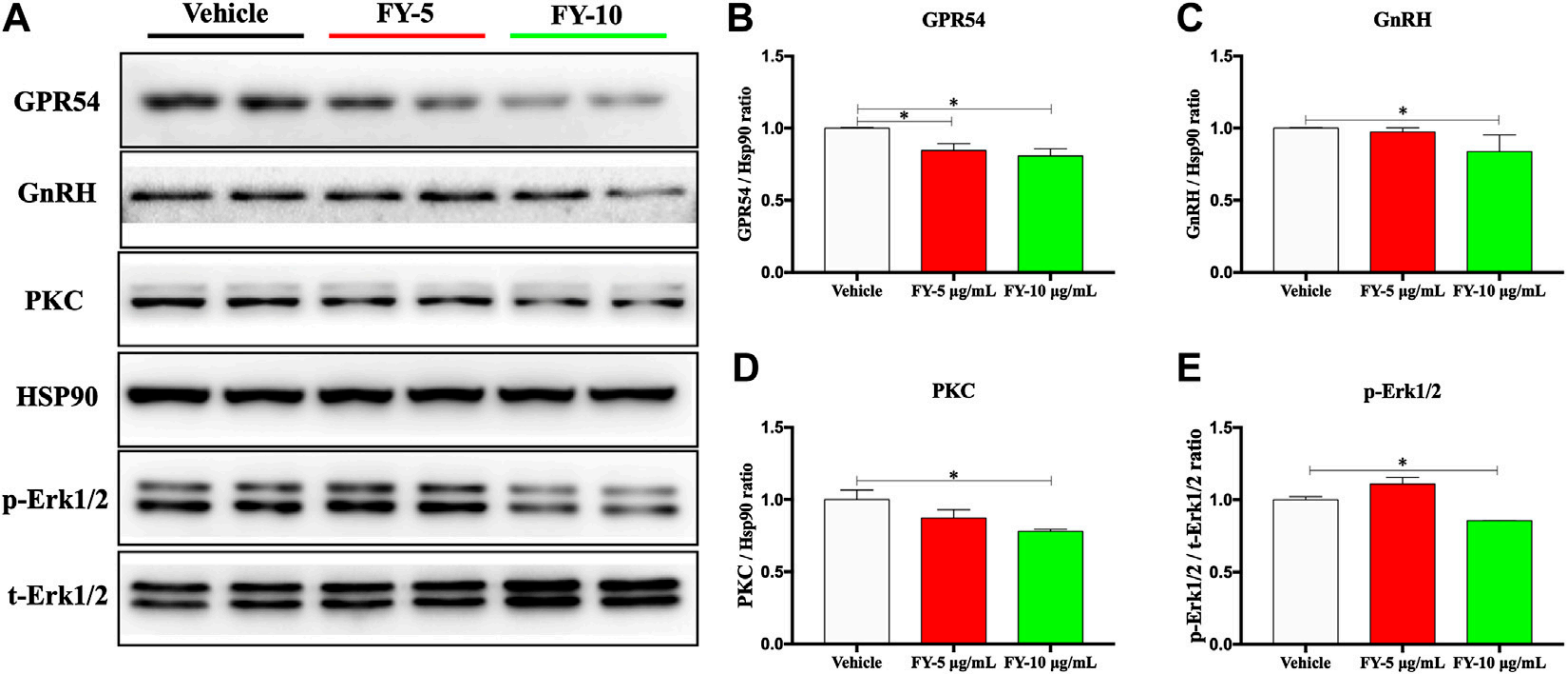
Effects of the Fy formula on GPR54, GnRH, PKC and pErk1/2 expression. **(A)** Representative images of western blots for GPR54, GnRH, PKC and pErk expression in GT1-7 cells after treatment with the Fy formula (5 or 10 μg/ml) for 24 h **(B–E)** Statistical analysis of GPR54, GnRH, PKC and pErk1/2 expression. *p < 0.05 vs. the vehicle group.

To additionally investigate the potential molecular mechanism underlying the effect of the Fy formula on the release of GnRH via the GPR54/GnRH signaling pathway, western blot analysis of the protein expression of GPR54, GnRH, PKC and pErk1/2 was conducted in GT1-7 cells treated with the Fy formula (5 or 10 μg/ml). GT1-7 cells expressed the GPR54 protein, as previously reported (Novaira et al., 2009b). We also performed western blot analysis of total and phosphorylated PKC and pErk1/2, as phosphorylation of these proteins is considered to be the main cellular signaling event in GPR54/GnRH activation. The protein levels of GnRH and PKC and the phosphorylation of Erk were decreased by incubation with Fy (10 μg/ml), as shown in Figures 5C–E. Compared with the vehicle, Fy formula at concentrations of 5 and 10 μg/ml decreased the protein expression of GPR54 (Figure 5B).

## Discussion

PP is defined as puberty that occurs before the age of 8 years in girls and before the age of 9 years in boys. PP has a far-reaching influence on children’s growth, development and mental health. Chinese herbal medicines have been proven to be effective in regulating the development of puberty, but their mechanisms are still unclear (Wang et al., 2014b; Yu et al., 2014). The GPR54/GnRH signaling pathway plays an important role in puberty development. The purpose of this study was to investigate the effects of the Fy formula on the GPR54/GnRH signaling pathway in GT1-7 cells and female rats with PP.

Danazol, an isoxazole derivative of 17α-ethynyl testosterone, has a variety of effects on the reproductive system, high affinity for androgen receptors, moderate affinity for glucocorticoid receptors and poor affinity for estrogen receptors. This study showed that subcutaneous injection of 300 µg of danazol into PND5 female rats accelerated the onset of sexual development, increased the weights of the uterus and ovary, increased the release of GnRH from the hypothalamus and accelerated HPG axis activation in rats, thus hastening sexual maturity without increasing the body of the rats (Morishita et al., 1993; Sun et al., 2010). These findings suggest that the rat hypothalamus is immature at birth, hypothalamic maturation may occur from 1 to 10 days after birth, and subcutaneous injection of danazol into newborn rats may quicken the maturation of the hypothalamus-pituitary axis and induce true PP (Sun et al., 2007).

Vaginal opening is a sign of puberty in female rats (Sun et al., 2007). Our experiment showed that the time of vaginal opening was significantly advanced in the model group compared with the control group (*p* < 0.001). After administration of leuprorelin and the Fy formula, vaginal opening was significantly delayed compared with that in the model group (*p* < 0.05, Figure 2A). The wet weights and organ coefficients of the uterus and ovary in the model group were significantly higher than those in the control group (*p* < 0.05) and were decreased after leuprorelin and Fy formula treatment (*p* < 0.05, Table 3). The fallopian tubes were thicker in the model group than the control group and returned to normal thickness after administration of the Fy formula. However, the fallopian tubes were significantly thinner in the leuprorelin group than the model group (Supplementary Figure S2), suggesting that leuprorelin affects the normal development of sex organs and should be combined with growth hormone (Benabbad et al., 2018). The Fy formula and leuprorelin decreased the serum levels of the hormones E2, LH and FSH compared with those in the model group, suggesting that the Fy formula can significantly delay puberty onset in female rats.

Although the physiological structure of humans is different from that of rodents, the Fy formula was shown to have therapeutic effects in both PP patients and a rat model of danazol-induced PP. As an in-hospital formula of Beijing Children’s Hospital, the Fy formula has been used for the management of PP for more than twenty years. These findings confirm the clinical benefits of the Fy formula, which is an advantageous therapeutic approach in our country due to its low cost. We enrolled patients who were treated for more than one year. The clinical data revealed that the Fy formula markedly reduced the serum levels of estrogen in patients, significantly decreased mammary gland size, and delayed bone maturation in girls with PP (Supplementary Table S1). Moreover, several Chinese reports also indicated that the Fy formula of Chinese herbs has a good intervention effect on girl’s ovarian cysts complicated with PP, causing most ovarian cysts to disappear (Pan Yu-chen and Liu, 2019). The clear clinical efficacy of the Fy formula is enough to prove its effective role in the treatment of PP in children. However, the physiological structure and function of humans and rats are different, so more evidence is needed to prove the mechanism of the Fy formula.

The research in humans and mice has shown that mutations or deletions in GPR54 lead to puberty failure and infertility, which suggests that the kisspeptin-GPR54 signaling pathway is absolutely essential for fertility (De Roux et al., 2003; Seminara et al., 2003; Clarkson, 2013). Subsequent research has shown that the loss of Kiss1 gene function in mice and humans results in pubertal disorders and infertility symptoms similar to GPR54 knockout (Topaloglu et al., 2012). Studies in the last decade have shown that kisspeptin and GPR54 are important regulators of GnRH activity. During puberty maturation, the increased expression of kiss1 mRNA and kisspeptin peptide in the hypothalamus is an important in promoting the full activation of GnRH nerve cells, and plays an important role in adolescent maturation and adult reproductive physiology (Clarkson, 2013), Therefore, kisspeptin mainly regulates reproductive events, such as precocious puberty, primarily by activating the GPR54 receptor indicated by GnRH neurons in the hypothalamus.

To further explore the role of GPR54/GnRH in the effects of the Fy formula on PP, gene expression was analyzed. Our results showed that kiss1, GPR54 and GnRH mRNA expression was higher in the model group than in the control group. One of our most obvious findings was that compared to vehicle, the Fy formula significantly downregulated the gene expression of the Kiss1, GPR54 and GnRH in the hypothalamus of female rats with PP (Figures 2E–G). Previous studies have shown that the Kiss1 gene is involved in the development of sexual maturity during early puberty in normal female rats (Sun et al., 2007; Sun et al., 2010) and that kisspeptin administration into the hypothalamus of immature female rats can significantly advance vaginal opening, significantly increase uterine weight (Navarro et al., 2004), and induce precocious activation of the HPG axis and PP development (Plant et al., 2006; Trevisan et al., 2018). These findings suggest that the Fy formula may inhibit the expression of the kiss1 gene, weaken the binding between kisspeptin and GPR54, and ultimately reduce the pulsed secretion of GnRH, weakening the activation of downstream pituitary and gonadal development and thus alleviating PP.

Kisspeptin and its cognate receptor GPR54 are major regulators of the HPG axis (Szereszewski et al., 2010). Kisspeptin is a neuropeptide encoded by the kiss1 gene and secreted in the hypothalamus. Kisspeptin has a highly conservative region at the C-terminal sequence, consisting of 10 amino acids, named kisspeptin-10 (Pasquier et al., 2014). GPR54 couples to the G_q/11_ protein when kisspeptin-10 stimulates GPR54, and G_q/11_ activates phospholipase C and hydrolyzes PIP2 to produce the second messengers IP3 and DAG. IP3 stimulates the endoplasmic reticulum (ER), increasing the levels of intracellular Ca^2+^ and activating Ca-dependent signaling pathways in GnRH neurons, and DAG activates protein kinase C (PKC), activates extracellular signal-regulated kinases one and two in GnRH neurons, and promotes hypothalamic secretion of GnRH (Kotani et al., 2001; Harter et al., 2018; Trevisan et al., 2018). The function of kisspeptin in GnRH neurons also requires the activation of additional signaling mechanisms in the family of MAPKs, which have a strong continuous stimulation effect on the phosphorylation of MAPK extracellular signal-regulating kinases Erk1 and Erk2 (Chianese et al., 2016). Kisspeptin is produced by two main groups of neurons located in the hypothalamus, namely, neurons in the rostral periventricular area of the third ventricle (RP3V) and neurons in the arcuate nucleus (ARC). Kisspeptin produced in the ARC is generally considered to be the generator of GnRH. GPR54 is mainly expressed in the hypothalamus, specifically the POA (Herbison et al., 2010), and is expressed by most GnRH-secreting neurons. Human GPR54 is highly expressed in the placenta, anterior pituitary, pancreas, liver, adipose tissue and spinal cord, suggesting that it plays a role in the regulation of endocrine function (Kotani et al., 2001; Ohtaki et al., 2001). In humans and rats, the onset of puberty is caused by an increase in GnRH pulsed secretion. True PP is mediated by a premature increase in GnRH secretion and should be treated with a potent GnRHa (Tian et al., 2005). The administration of exogenous kisspeptin can advance the puberty of immature rodent (rats) and primates (monkeys) (Plant et al., 2006), on the contrary, administration of kisspeptin antagonist delayed puberty in rats and monkeys (Skorupskaite et al., 2014; Li et al., 2020). These findings show that kisspeptin plays an important role in the regulation of HPG axis.

**FIGURE 6:**
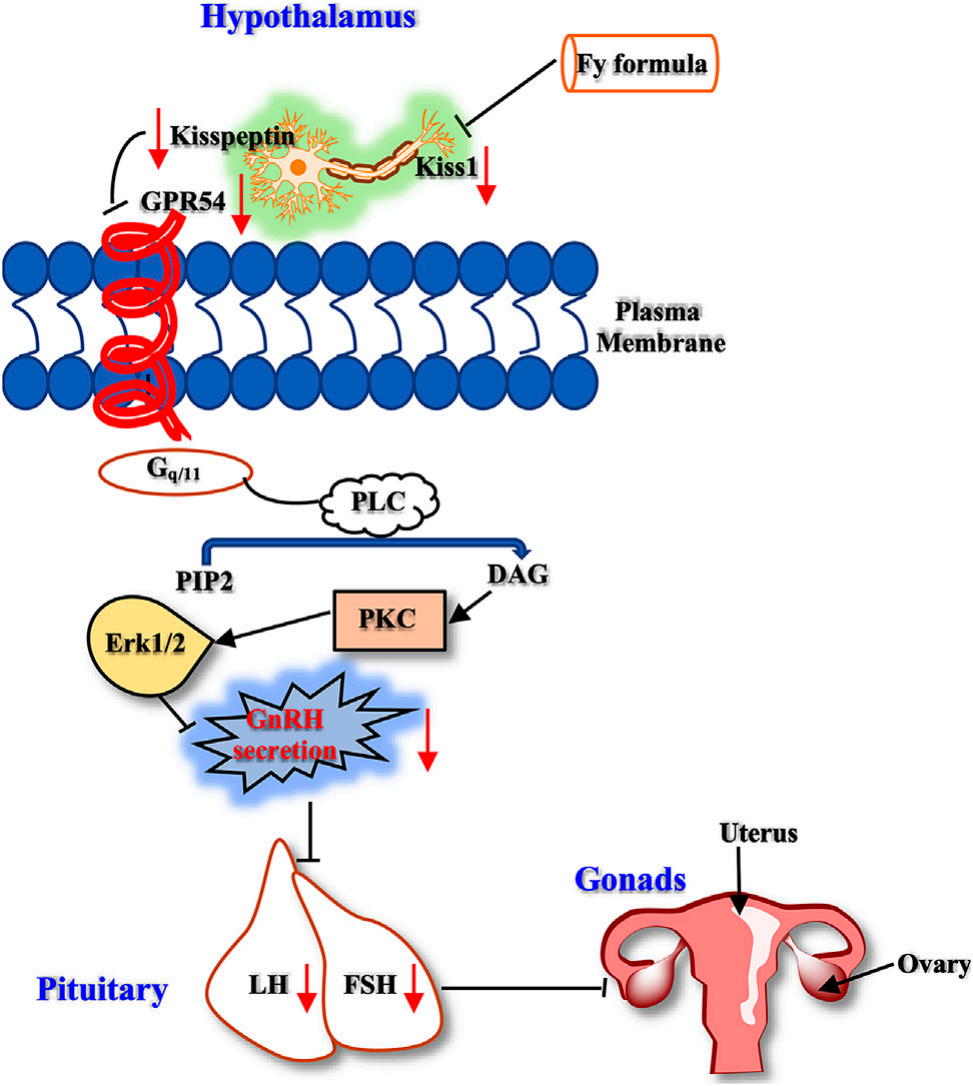
The GPR54/GnRH signaling pathway in the HPG axis. The Fy formula inhibits the gene expression of Kiss1, decreases the secretion of kisspeptin, and weakens the stimulating effect on the kisspeptin receptor GPR54. Gq/11 inhibits PLC, which reduces the hydrolysis of PIP2. This process leads to a decrease in the production of the second messenger DAG, which inhibits the downstream Erk1/2 signaling pathway, resulting in a decrease in GnRH secretion, weakening the stimulatory effect on the pituitary gland, reducing the secretion of LH and FSH, and inhibiting gonadal development.

Therefore, kisspeptin mainly regulates reproductive events, such as precocious puberty, primarily by activating the GPR54 receptor indicated by GnRH neurons in the hypothalamus. In the present study, we designed a series of experiments to investigate the mechanism of the Fy formula. First, we determined that kisspeptin at a concentration of 10^−9^ M had a direct effect on GT1-7 cells and upregulated the expression of the GPR54, GnRH, Erα and Erβ genes (Figure 4; Supplementary Figure S2). These results are consistent with previous reports showing that which kisspeptin can promote the GnRH system (Clarkson et al., 2008; Novaira et al., 2009a). Kisspeptin binds to GPR54 and stimulates hypothalamic neurons to release GnRH, leading to the secretion of gonadotropins (LH and FSH) and sex steroids, which in turn act on the gonads to produce gametes, by the pituitary gland (Trevisan et al., 2018). The Fy formula (10 µg/ml) decreased the gene expression of GPR54, GnRH, Erα and Erβ, which was increased by kisspeptin in GT1-7 cells (Figures 4A–D). Kisspeptin-10 not only upregulated the expression of the GnRH gene but also increased the secretion of GnRH in GT1-7 cells (Figure 4E), which is consistent with previous results (Lehnert and Khadra, 2019). However, after administration of the Fy formula, the secretion of GnRH was significantly decreased (Figure 4E). To confirm the effect of the Fy formula on the GPR54/GnRH signaling pathway in GT1-7 cells, we investigated its effects on related proteins and found that the Fy formula significantly downregulated the protein expression of GPR54, PKC and GnRH and the phosphorylation of Erk1/2 (Figure 5). These results indicate that the Fy formula inhibits Kiss1 gene expression, reduces the expression of kisspeptin, reduces the binding of GPR54 to Gq/11, inhibits the hydrolysis of PIP2 and the Erk1/2 signaling pathway, decreases the secretion of hypothalamic GnRH, weakens the stimulation of the pituitary gland, reduces the secretion of the gonadotropins LH and FSH and sex steroids, inhibits gonadal gamete production and alleviates the symptoms of PP (Figure 6) (Roseweir and Millar, 2009; Trevisan et al., 2018).

In this study, the therapeutic effect of the Fy formula on PP was studied in GT1-7 cells and in a rodent model. Additionally, it has also been observed clinically that the Fy formula can reduce the estrogen level in girls with PP, significantly decreased mammary gland size, and delayed bone maturation. However, because the clinical sample size in this study was small, the data may have been slightly skewed. Therefore, it is necessary to increase the clinical sample size and evaluate clinical indicators at a later stage to more comprehensively assess the curative effect of the Fy formula.

## Conclusion

In summary, the Fy formula reduced blood hormone levels, downregulated the gene expression of kiss1, GPR54 and GnRH in female rats with danazol-induced PP and downregulated GnRH gene and protein expression and hormone secretion induced by kp-10 in GT1-7 cells. The GPR54/GnRH signaling pathway may be involved in the effects of the Fy formula on PP. The potential mechanism of action of the Fy formula needs to be further revealed to provide support for its clinical application.

## Data Availability Statement

The original contributions presented in the study are included in the article/Supplementary Material, further inquiries can be directed to the corresponding authors.

## Ethics Statement

The studies involving human participants were reviewed and approved by Ethics Committee of Beijing Children’s Hospital (IEC-C-008-A08-V.05.1). Written informed consent to participate in this study was provided by the participants' legal guardian/next of kin. The animal study was reviewed and approved by Animal Ethics Committee of Capital Medical University (AEEI-2020-084). Written informed consent was obtained from the individual(s), and minor(s)' legal guardian/next of kin, for the publication of any potentially identifiable images or data included in this article.

## Author Contributions

GB performed the investigation and wrote the paper. KH, YH, XW, LL and HC participated in the collection and preparation of rat samples. MZ, CG and JL participated in the collection of patients data. LZ, ZS, XW, and XN designed the study and revised the paper. All authors reviewed and approved the final manuscript.

## Funding

This work was supported by the National Major Science and Technology Projects of China, China (grant no. 2018ZX09721003) and the Capital Special Health Development Research (grant no. 2018-2-2097).

## Conflict of Interest

The authors declare that the research was conducted in the absence of any commercial or financial relationships that could be construed as a potential conflict of interest.

The reviewer PZ declared a shared affiliation with the authors to the handling editor at the time of the review.
