# Supplementary material for: The Traditional Chinese Medicine Fuyou Formula Alleviates Precocious Puberty by Inhibiting GPR54/GnRH in the Hypothalamus
Source: Front Pharmacol. 2021 Jan 21;11:596525. doi: 10.3389/fphar.2020.596525 (PMC7859969; doi:10.3389/fphar.2020.596525)
Supplement: Supplementary file 1 [file table1.docx]

***Supplementary materials for*：****The Traditional Chinese Medicine Fuyou Formula Alleviates Precocious Puberty by Inhibiting GPR54/GnRH in the Hypothalamus**

Guo-liang Bai^1, 2^, Kai-li Hu^3^, Yi Huan^2^, Xing Wang^2^, Lei Lei^2^, Meng Zhang^1^, Chun-yan Guo^1^, Hongsheng Chang^3^, Libo Zhao^1^, Jing Liu^1^, Zhu-fang Shen^2*^, Xiao-ling Wang^1*^, Xin Ni^1*^

^1^ Clinical Research Center, Beijing Children’s Hospital, Capital Medical University, National Center for Children’s Health, Beijing 100045, China

^2^ State Key Laboratory of Bioactive Substances and Functions of Natural Medicines, Key laboratory of Polymorphic Drugs of Beijing, Institute of Materia Medica, Chinese Academy of Medical Sciences, Peking Union Medical College, Beijing 100050, China

^3^ Department of Pharmacology, School of Chinese Materia Medica, Beijing University of Chinese Medicine, Beijing 100102, China

**Correspondence:**Zhu-fang Shen, e-mail: [shenzhf@imm.ac.cn](mailto:shenzhf@imm.ac.cn); Xiao-ling Wang, e-mail:[eyjdb6380@163.com](mailto:eyjdb6380@163.com); Xin Ni, e-mail: [nixin@bch.com.cn](mailto:nixin@bch.com.cn), Postal address: Beijing Children’s Hospital, Capital Medical University, National Center for Children’s Health, 56 Nanlishi Road, Xicheng District, Beijing 100045, China

* To whom correspondence should be addressed

***1.1 Cytotoxicity.***

The cell viabilitiy of Fy formula was compared with that of the DW group on the GT1-7 cells. The maximum non-cytotoxic concentration of Fy formula was 10 ug/mL (Figure. S1), in order to ensure the efficacy of the drug, the final treatment concentration of all subsequent treatments of GT1-7 cells were 5 ug/mL and 10 ug/mL.


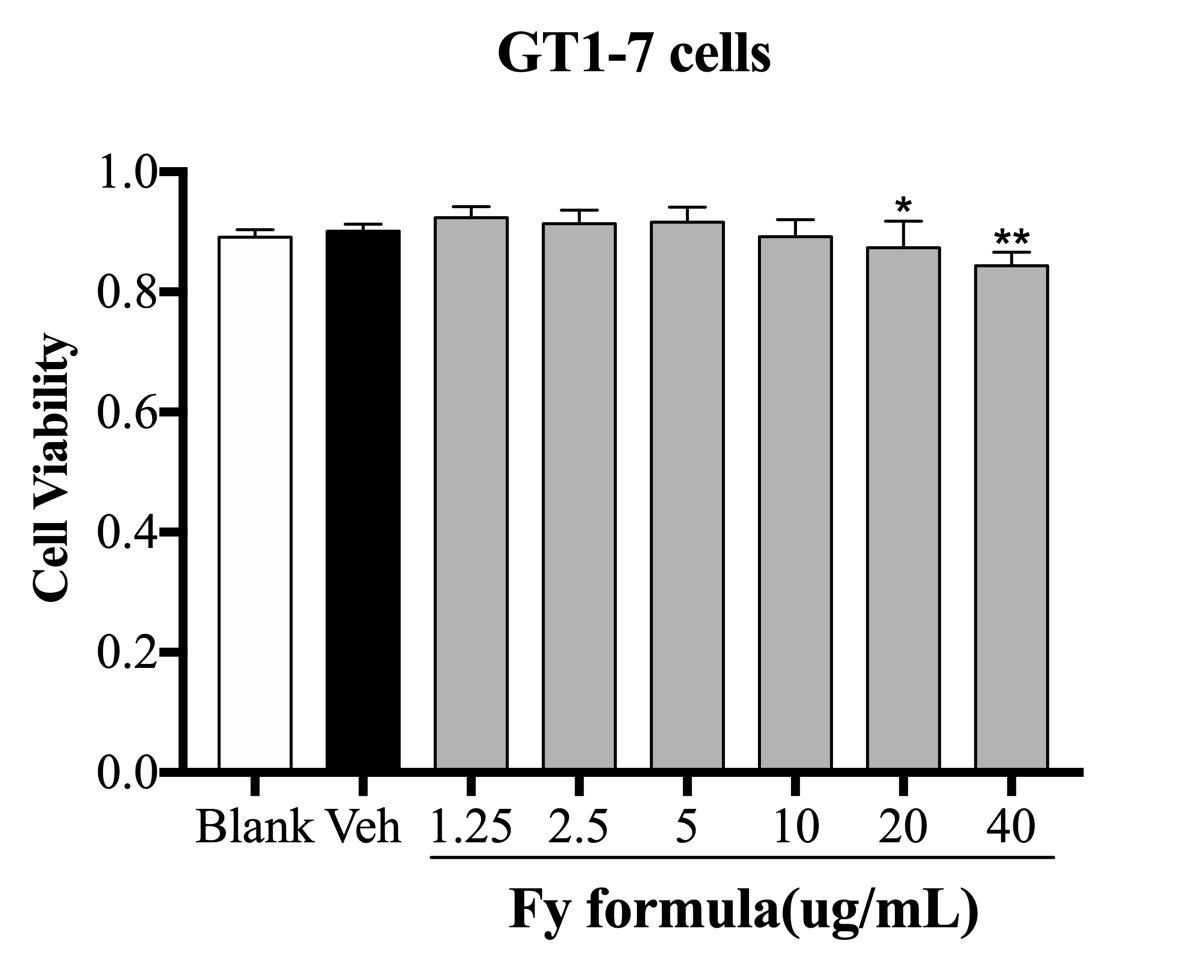


**FIGURE S1|** Cell viability of GT1-7 cells treatment with Fy formula for 24 h was analyzed by CCK-8. The results ran in sextuplicate and expressed as mean ± SD of OD 450 nm, statistical analysis of Fy formula was compared to Vehicle (distilled water treated cells). *p < 0.05, **p < 0.01, ***p < 0.001vs Vehicle.

***1.2 GnRH mRNA expression is increased by kp-10 in GT1-7 cells***

Changes in GnRH mRNA was evaluated by RT-PCR which treating with different concentrations of kp-10 in GT1-7 cells for 4 h and 24 h respectively. The expression of GnRH mRNA was the highest treated with kp-10 (10^-9^ M) in GT1-7 cells, which was 1.6 times compared with the Vehicle group (n=5, p < 0.01, Fig. S2A), while the level of GnRH mRNA was 4.2 fold higher compared with Vehicle group after 24 h treatment which treated with 10^-9^ M kp-10 (n = 5, p < 0.001, Fig. S2B). Therefore, the expression of GnRH mRNA was the highest when the GT1-7 cells were treated with kp-10 at 10^-9^ M for 24 h.


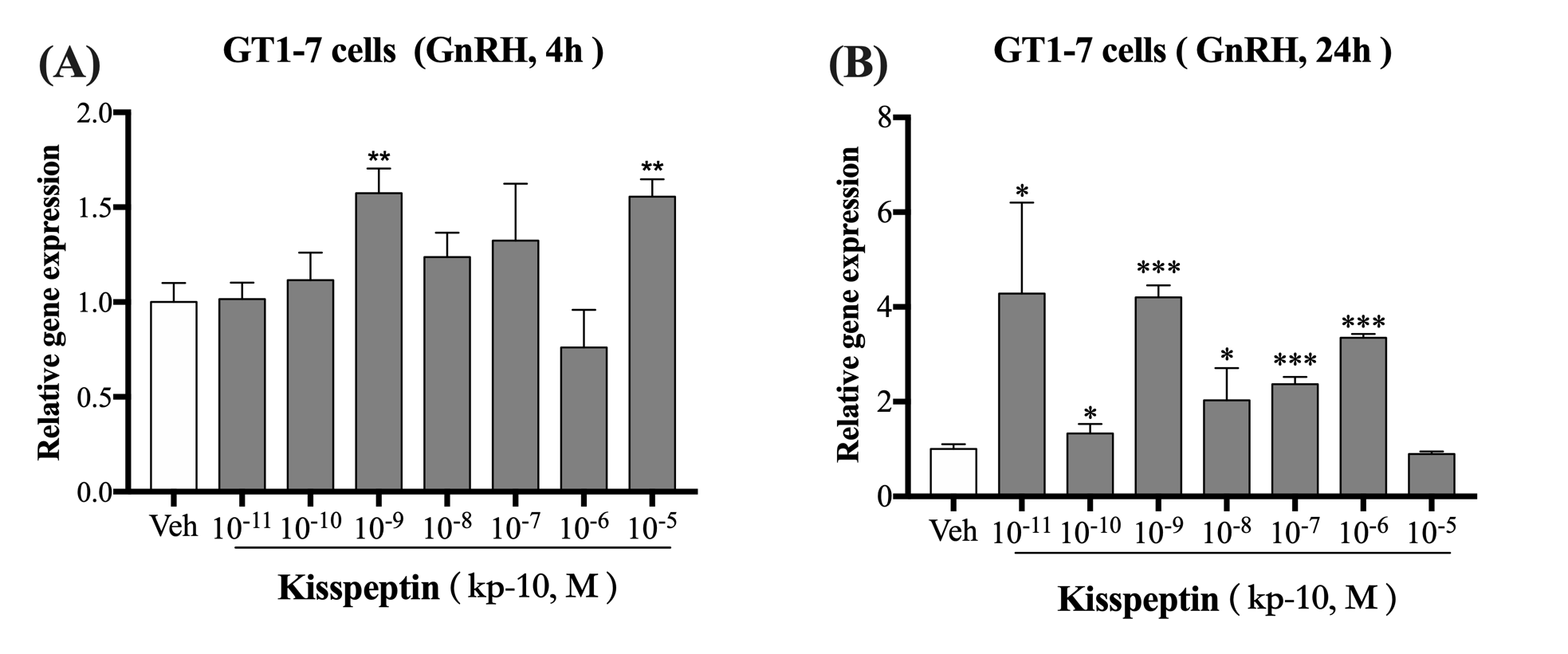


**FIGURE S2|**The dose-response and time-course of GnRH mRNA expression by kp-10 in GT1-7 cells. (B-C): Gene expressions of GnRH in GT1-7 cells treated with of kp-10 (concentration from 10^-11^ to 10^-5^ M) for 4h and 24h were analyzed by Real-time PCR. *p < 0.05, **p < 0.01 vs Vehicle

***1.3 Fy formula alleviates the clinical symptoms of PP in girls***

A total of 20 patients who came to Beijing Children’s Hospital from January 2017 to January 2019 were enrolled and treated for approximately one year. The serum level of the hormone E2 decreased from 40.485 ± 14.127 pmol/L before treatment to 27.380 ± 7.676 pmol/L after treatment (p < 0.001). Fy formula, significantly decreased left and right mammary gland size and obviously delayed bone development (p < 0.001, Table 1). The above results show that Fy formula improves the clinical symp- toms of PP in girls.

Table S1. Clinical features of girls with PP before and after treatment with Fy formula.

|  | Group | Mean ± SD | p value |
| --- | --- | --- | --- |
| E2 (pmol/L) | Before treatment | 40.49 ± 14.13 | p = 0.0008 |
|  | After treatment | 27.38 ± 7.68 |  |
| Left mammary gland size (cm) | Before treatment | 2.9 ± 0.68 | p = 0.0002 |
|  | After treatment | 1.2 ± 0.23 |  |
| Right mammary gland size (cm) | Before treatment | 2.6 ± 1.12 | p = 0.0183 |
|  | After treatment | 1.2 ± 0.48 |  |
| Bone age (△BA/△CA) | Before treatment | 1.34 ± 0.43 | p = 0.0001 |
|  | After treatment | 1.27 ± 0.35 |  |
